# Successful Implantation of Rapid Deployment Aortic Valve after TAVR Explantation

**DOI:** 10.1186/s13019-024-02728-5

**Published:** 2024-04-15

**Authors:** Yusuke Yanagino, Naonori Kawamoto, Satoshi Kainuma, Naoki Tadokoro, Takashi Kakuta, Ayumi Ikuta, Kohei Tonai, Tomoyuki Fujita, Satsuki Fukushima

**Affiliations:** Department of Cardiovascular Surgery, National Cerebral and Cardiovascular Center, 6-1 kishibe- shimmachi, Suita, 564-8565 Osaka Japan

**Keywords:** Transcatheter aortic valve replacement, Rapid deployment valve, Prosthetic valve dysfunction

## Abstract

**Background:**

Transcatheter aortic valve replacement (TAVR) has become widely used in recent years, However, there is also an increasing need for removal of TAVR valves due to prosthetic valve dysfunction (PVD) and the development of infective endocarditis. Surgical aortic valve replacement (AVR) for these patients is risky due to the original patient background and anatomic conditions. Intuity rapid deployment aortic valve (Edwards Lifesciences, Irvine, CA) replacement would be useful for such high risk patients to prevent longer cardiac arrest time and obtain good hemodynamic results. However, there are few reports which present Intuity valve replacement after TAVR explantation. Herein, We report two cases in which we have achieved good hemodynamics with shorter cardiac arrest times by using a rapid deployment valve after TAVR explantation.

**Case presentation:**

We present 2 cases of successful implantation of the Intuity rapid deployment valve after TAVR explantation. The 84- and 88-year-old female patients had previously received TAVR for severe aortic stenosis with SAPIEN XT (Edwards Lifesciences, Irvine, CA) and developed PVD during follow-up. The TAVR valve was removed carefully, then an Intuity valve was implanted with cardiac arrest times of 69 and 41 min. Both patients had good echocardiographic results with effective orifice area of 2.0 cm^2^ and 1.2 cm^2^ and mean trans-aortic plessure gradient of 9 mmHg and 15 mmHg respectively without aortic regurgitation. They were discharged without major complications.

**Conclusions:**

Surgical AVR using a rapid deployment valve is a useful alternative to sutured AVR after TAVR valve explantation. It allows for shorter cardiac arrest times and better postoperative hemodynamics without major complication.

## Backgrounds

The indications for transcatheter aortic valve replacement (TAVR) have rapidly expanded from high-risk to intermediate- and even low-risk patients. With the increasing number of TAVR procedures, the demand for TAVR valve explantation for prosthetic valve deterioration (PVD) is expected to increase in the coming years. TAVR valve explantation and aortic valve replacement (AVR) reoperation can be technically challenging due to significant device incorporation into the surrounding cardiac structures. TAVR-in-TAVR procedures are an effective option that could be used as an alternative to conventional AVR. However, there are some patients in whom TAVR-in-TAVR is not feasible because of infection and unfavorable anatomy. Rapid deployment AVR can reduce arrest time and provide better hemodynamic performance than sutured AVR, and therefore, might be another valid option for PVD after TAVR [1]. Here we report 2 octogenarian patients who presented with PVD after TAVR with SAPIEN XT (Edwards Lifesciences, Irvine, CA) and underwent successful complication-free explantation of the SAPIEN XT valve and surgical AVR reintervention using an Intuity rapid deployment valve (Edwards Lifesciences, Irvine, CA).

## Case presentation

### Patient 1

An 84-year-old woman presented with dyspnea 4 years after undergoing TAVR with a 26-mm SAPIEN XT bioprosthetic valve for severe aortic stenosis. Echocardiography revealed severe PVD with a mean pressure gradient of 83 mmHg, aortic valve area of 0.61 cm [2], peak velocity of 5.6 m/s, and moderate perivalvular aortic regurgitation (PVL). Although the patient was at very high risk of an adverse outcome with conventional AVR (The Society of Thoracic Surgeons (STS) score 12.5%), TAVR explant and AVR reintervention using an Intuity rapid deployment valve were scheduled.

**Fig. 1:**
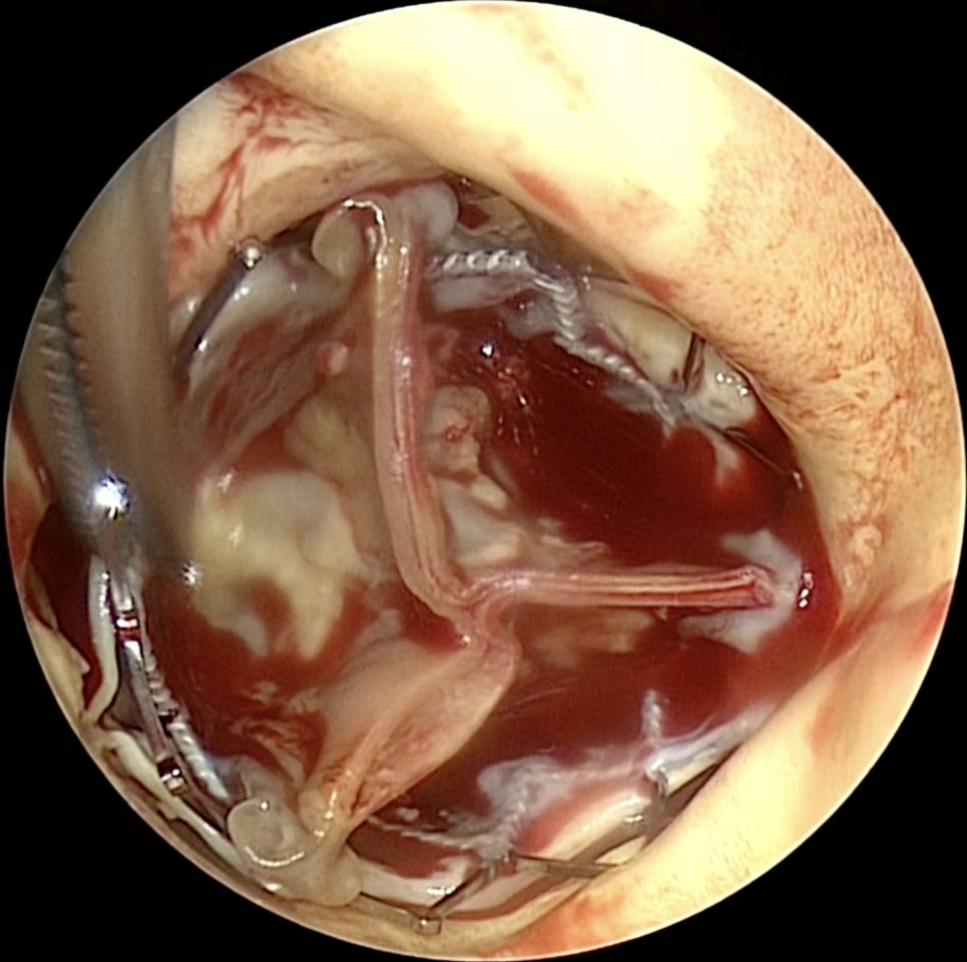
The SAPIEN XT valve in aorta SAPIEN XT valve was severely adhered to the aortic annulus and surrounding structures

**Fig. 2:**
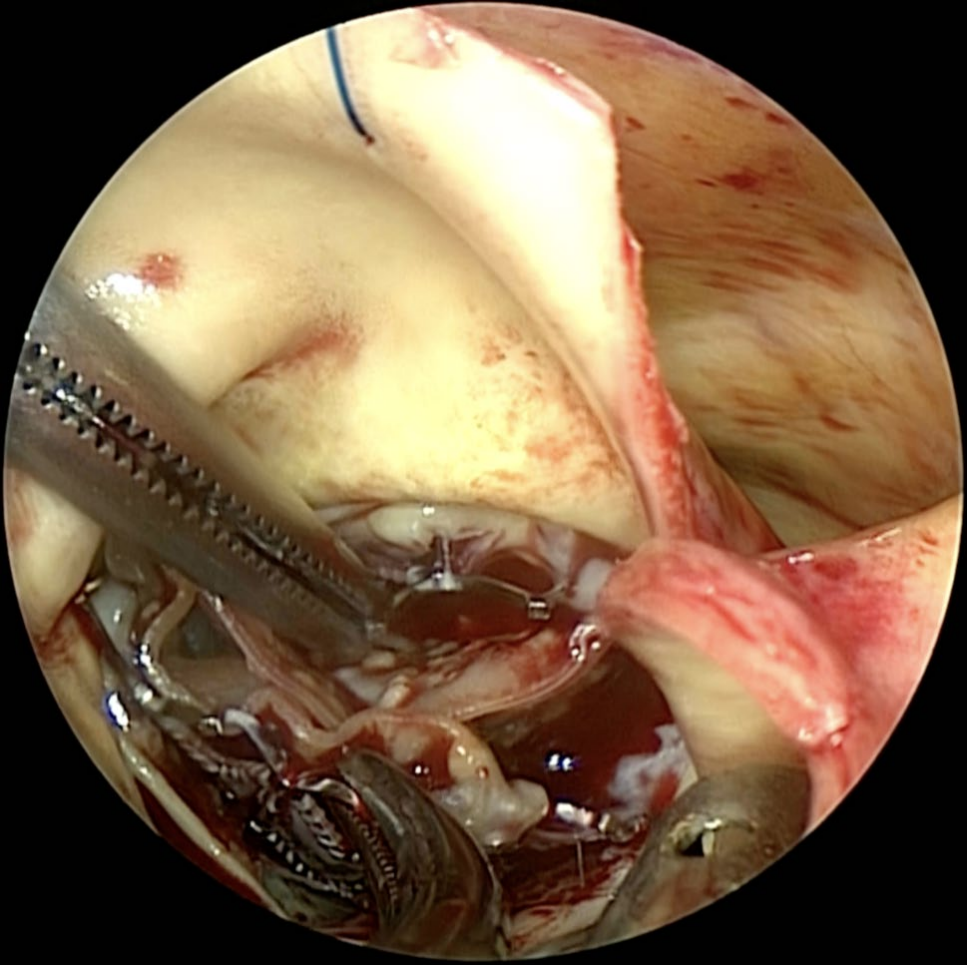
The double Kocher technique The double Kocher technique was feasible to preserve aortic root structure in TAVR explantation

**Fig. 3:**
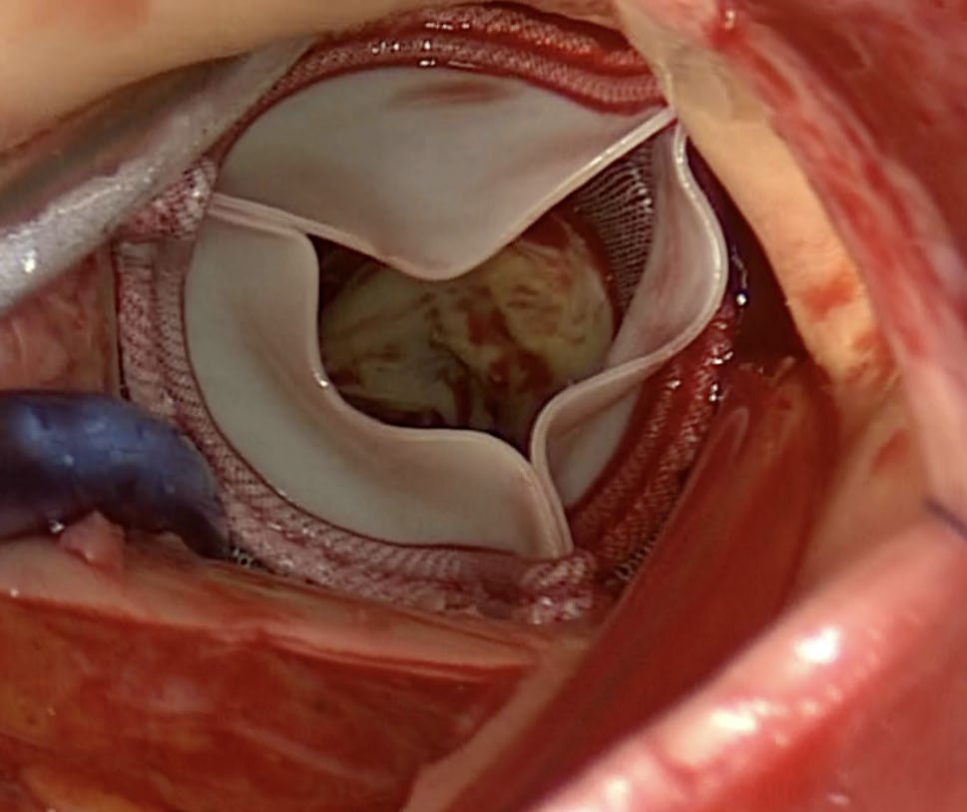
Aortic valve replacement using a 23-mm Intuity rapid deployment bioprosthetic valve The Intuity rapid deployment valve was well-seated in the aortic annulus without peri-valvular leakage

The surgery was performed through median sternotomy under standard cardiopulmonary bypass support. After cardiac arrest, a transverse aortotomy was made to expose the TAVR valve, which was neoendothelialized and incorporated into the aortic root (Fig. 1). After making the endarterectomy plane between the aorta and the stent frame, the plane was carefully extended to avoid native tissue. Using the double Kocher Clamp technique [2], two forceps were applied to push the stent frame towards the center away from native tissue and avoid injuring the anterior mitral leaflet and coronary ostia (Fig. 2). The explanted valve showed significant calcification of the cusps and reduced flexibility. Native aortic cusps and calcified annulus that had been compressed by the TAVR valve were prudently excised. Aortic valve replacement was performed using a 23-mm Intuity rapid deployment bioprosthetic valve to reduce the cardiac arrest time and obtain better hemodynamics [1] (Fig. 3).

Cardiopulmonary bypass time and cardiac arrest time were 103 and 69 min, respectively. There were no major complications, including conduction disturbances, after the surgery. Postoperative echocardiography demonstrated a preserved left ventricular ejection fraction of 59%, a mean trans-aortic pressure gradient of 9 mmHg, an effective orifice area of 1.97 cm [2], and no aortic regurgitation. The patient suffered symptomatic paroxysmal atrial fibrillation after surgery, which prevented her recovering from postoperative disuse syndrome. Finally, she was discharged to a rehabilitation hospital on postoperative day 59.

### Patient 2

An 88-year-old woman presented with dyspnea 6 years after undergoing TAVR with a 23-mm SAPIEN XT bioprosthetic valve for severe aortic stenosis. Echocardiography revealed severe PVD with a mean pressure gradient of 49 mmHg, aortic valve area of 0.6 cm [2], and peak velocity of 4.6 m/s. Although the patient was at high risk for adverse outcomes with conventional AVR (STS risk score 8%), TAVR explant and AVR reintervention using an Intuity rapid deployment valve were scheduled.

The surgery was performed using the same method as described for the first patient. Aortic valve replacement was performed using a 21-mm Intuity rapid deployment bioprosthetic valve.

Cardiopulmonary bypass time and cardiac arrest time were 76 and 41 min, respectively. There were no major post-surgical complications, including conduction disturbances. Postoperative echocardiography demonstrated a preserved left ventricular ejection fraction of 64%, a mean trans-aortic pressure gradient of 15 mmHg, an effective orifice area of 1.2 cm [2], and no aortic regurgitation. The patient was complicated by disuse syndrome, but finally discharged on postoperative day 41.

## Discussion and conclusions

TAVR was first approved in 2002 as an alternative treatment for severe aortic stenosis in high-risk surgical patients. TAVR is now available for lower-risk or younger patients. As the use of TAVR extends to younger and lower-risk populations, the demand for TAVR explant due to PVD or other reasons, including infection, will increase rapidly. However, previous reports showed that TAVR explant might be associated with worse-than-expected 30-day mortality [3]. In one of those reports, although the STS mortality risk at the time of TAVR explant was 5%, the observed 30-day postoperative mortality was 13%. Neoendothelialization and severe adhesion to surrounding tissue can make it technically challenging to excise the transcatheter heart valve without injury to native tissue. Moreover, aortic root replacement was needed for approximately 10% of patients with balloon-expandable TAVR [3]. Longer aortic cross-clamp time and cardiopulmonary bypass (CPB) time might be associated with higher mortality [4].

Conventional sutured AVR using stented prosthesis is the standard technique after TAVR explant. However, rapid deployment AVR, including the Intuity valve system, was highly effective in reducing CPB time and cardiac arrest time, compared with conventional sutured AVR. International Registry (SURD-IR) reported about 30 min shorter CPB and closs-clamp time compaired with the Society of Thoracic Surgeons (STS) conventional AVR data base (79 and 51 vs. 106 and 78 min) despite including minimum-invasive surgery in SURD-IR group [5]. The Intuity valve was also associated with good short-and mid-term outcomes and larger effective orifice areas, preventing patient-prosthesis mismatch, especially for a small annulus [1]. In redo AVR situation, there are a few articles [6, 7] related with use of sutureless AVR, especially after TAVR explant.

In our cases, cardiac arrest times were 69 min in patients 1 and 41 min in patient 2, which is shorter than previous reports of TAVR explant [3]. Furthermore, we had good hemodynamic results without major complications. While PVL and conduction disturbances may be a considerable issue after the use of rapid deployment AVR [8], we previously reported excellent early outcome of intuity valve replacement, which showed, which showed same PVL occurrence, no migration, and low PMI rate as conventional AVR in matched cohort [9]. In our cases, we could successfully remove the TAVR valve with preservation of the aortic root. This made it possible to identify the annulus and precisely deploy the Intuity rapid deployment valve without causing PVL or conduction disturbances. Even if intuity valve was not suitable after TAVR explant, sutured AVR would be possible from same aortotomy.

In conclusion, rapid deployment AVR was feasible and effective to treat PVD after TAVR, with relatively short cardiac arrest times and good post-procedure hemodynamic performance, and therefore, may be a valid option after late TAVR explantation.

## Data Availability

Data sharing is not applicable to this article as no datasets were generated or analysed during the current study.

## Abbreviations

TAVR: transcatheter aortic valve replacement
PVD: prosthetic valve dysfunction
AVR: aortic valve replacement
PVL: perivalvular aortic regurgitation
STS: The Society of Thoracic Surgeons
CPB: cardiopulmonary bypass
